# Lipoprotein-Associated Phospholipase A2 Activity and Mass as Independent Risk Factor of Stroke: A Meta-Analysis

**DOI:** 10.1155/2019/8642784

**Published:** 2019-05-20

**Authors:** Gaifeng Hu, Deping Liu, Huiyu Tong, Weijun Huang, Yunzhao Hu, Yuli Huang

**Affiliations:** ^1^Department of Cardiology, Shunde Hospital of Southern Medical University, Foshan 528300, China; ^2^Department of Cardiology, Beijing Hospital, National Center of Gerontology, No.1 DaHua Road, Dong Dan, Graduate School of Peking Union Medical College, Beijing, 100730, China

## Abstract

**Background:**

The association between lipoprotein-associated phospholipase A2 (Lp-PLA2) and stroke risk is inconsistent. We conducted a meta-analysis to determine whether elevated Lp-PLA2 is a risk factor for stroke.

**Methods:**

Studies were included if they reported Lp-PLA2 mass and/or activity levels and adjusted risk estimates of stroke. The primary outcome was overall stroke incidence. The combined results were shown as relative risks (RRs) with 95% confidence intervals (CI) for per 1 standard deviation (SD) higher value of Lp-PLA2 and the highest versus lowest Lp-PLA2 category.

**Results:**

Twenty-two studies involving 157,693 participants were included for analysis. After adjusting for conventional risk factors, the RRs for overall stroke with 1 SD higher Lp-PLA2 activity and mass were 1.07 (95% CI 1.02–1.13) and 1.11 (95% CI 1.04–1.19), respectively. The RRs of ischemic stroke with 1 SD higher Lp-PLA2 activity and mass were 1.08 (95% CI 1.01–1.15) and 1.11 (95% CI 1.02–1.22), respectively. When comparing the highest and lowest levels of Lp-PLA2, the RRs of stroke for Lp-PLA2 activity and mass were 1.26 (95% CI 1.03–1.54) and 1.56 (95% CI 1.21–2.00), respectively. Finally, when comparing the highest and lowest levels of Lp-PLA2, the pooled RRs of ischemic stroke for Lp-PLA2 activity and mass were 1.29 (95% CI 1.07–1.56) and 1.68 (95% CI 1.12–2.53), respectively.

**Conclusions:**

Elevated baseline Lp-PLA2 levels, detected either by activity or mass, are associated with increased stroke risk.

## 1. Introduction

Stroke results in 9% of all deaths and is the primary cause of disability around the world [1]. Tailored treatment based on risk factors may help to target therapies to high-risk patients and reduce stroke morbidity. Biomarkers are being investigated to improve stroke diagnosis and determine its cause.

Lipoprotein-associated phospholipase A2 (Lp-PLA2) is an enzyme mainly produced by macrophages and monocytes [2] and can hydrolyze oxidized phospholipids, produce oxidative modification of low-density lipoprotein, and release pro-inflammatory and pro-atherogenic metabolites. Thus, Lp-PLA2 may contribute to the development of atherosclerosis and plaque rupture, which in turn leads to coronary heart disease and stroke [3].

Although the US Food and Drug Administration approved a Lp-PLA2 blood test for assessing patients at risk for ischemic stroke in 2005, research into the association between Lp-PLA2 and stroke risk has yielded inconsistent results. The association of circulating Lp-PLA2 with ischemic stroke is less clear than with coronary heart disease, perhaps because fewer outcomes in ischemic stroke are recorded. Levels of Lp-PLA2 are measured in two ways: Lp-PLA2 activity and mass assays. Although Lp-PLA2 activity correlates with Lp-PLA2 mass [4], the risk of ischemic stroke with Lp-PLA2 activity and mass is inconsistent; for example, a meta-analysis of individual patient data (IPD) demonstrated that higher Lp-PLA2 mass, but not activity, may increase ischemic stroke risk [5]. More recently, however, increasing numbers of studies have reported an association between Lp-PLA2 and stroke risk. We conducted a meta-analysis to evaluate the association between baseline levels of Lp-PLA2 activity/mass and stroke risk.

## 2. Methods

### 2.1. Literature Search

This study was conducted according to the Preferred Reporting Items for Systematic Reviews and Meta-Analyses (PRISMA) statement [6] (Supplement 1). We searched relevant studies in PubMed, Embase, and Cochrane Library to February 2019. There were no restrictions on language or the publication date. Relevant studies were identified using following terms: “Lipoprotein-Associated Phospholipase A2” OR “1-Alkyl-2-acetylglycerophosphocholine Esterase” OR “Lp-PLA2” AND “stroke” OR “cardiovascular disease” OR “cerebrovascular disorder” OR “cardiocerebrovascular disease” OR “cardiovascular event” OR “cerebrovascular disease” OR “cerebrovascular attack” OR “cerebral infarction” OR “intracranial hemorrhage” OR “intracerebral hemorrhage” OR “Transient Ischemic Attack”. The detailed search strategy is presented in Online Supplement 2.

### 2.2. Study Selection Criteria

Studies were included if (1) they were prospective cohort studies (reported as observational cohort studies or case–cohort subsets) or randomized controlled trials with data on Lp-PLA2 mass and/or activity at baseline; (2) multivariate-adjusted relative risk ratios (RR) of stroke or transient ischemic attack (TIA) associated with Lp-PLA2 mass and/or activity were reported; and (3) participants were aged ≥ 18 years.

Studies were excluded if (1) they were retrospective or cross-sectional studies, (2) RR was adjusted for age and sex only, (3) RR with 95% confidence intervals (95% CI) were reported from only two quantitative exposure categories, or (4) data were derived from the same cohort or from combined analysis of other studies.

### 2.3. Data Extraction and Quality Assessment

Two authors (Gaifeng Hu and Deping Liu) extracted relevant information from the eligible studies independently. Any divergences were resolved by discussion. Study quality was independently assessed by two authors (Huiyu Tong and Weijun Huang) conforming to the Newcastle Ottawa Scale (NOS) [7]. Studies getting a rating of ≥ 8 stars on the NOS indicated the high study quality; all others were considered as low or moderate quality.

### 2.4. Pooled Data Analysis

The primary outcome was stroke, including ischemic stroke, hemorrhagic stroke, unclassified stroke, and TIA. The secondary outcome was ischemic stroke. Subgroup analyses of the primary outcome were conducted according to sex, age, study design, follow-up period, inclusion of participants with baseline cardiovascular disease (CVD; yes vs. no), inclusion of participants with baseline CVD type, ischemic stroke event sizes, and NOS quality.

Multivariate-adjusted RR or hazard ratios with 95% CI were used for analysis. The inverse variance approach was used to combine the log RR and SE values.

We performed two comparisons. First, we compared stroke risk in individuals with the highest quantile of Lp-PLA2 with those with the lowest quantile of Lp-PLA2. Second, we calculated the RR for a 1-standard-deviation (SD) change in baseline levels of Lp-PLA2 mass and activity.

We used *χ*^2^ and I^2^ statistics to examine heterogeneity. Values of* P* < 0.10 or I^2^ > 50% indicated significant heterogeneity across studies. We also reported results from random-effects models. Publication bias was assessed by Begg's test, and a sensitivity analysis was conducted by omitting one study at a time.

Statistical analyses were conducted using RevMan software (v5.3 for Windows, The Cochrane Collaboration, Copenhagen, Denmark) and Stata (v13.0, Stata Corp LP, College Station, Texas). A value of* P* < 0.05 was considered statistically significant.

## 3. Results

### 3.1. Study Characteristics

**Figure 1:**
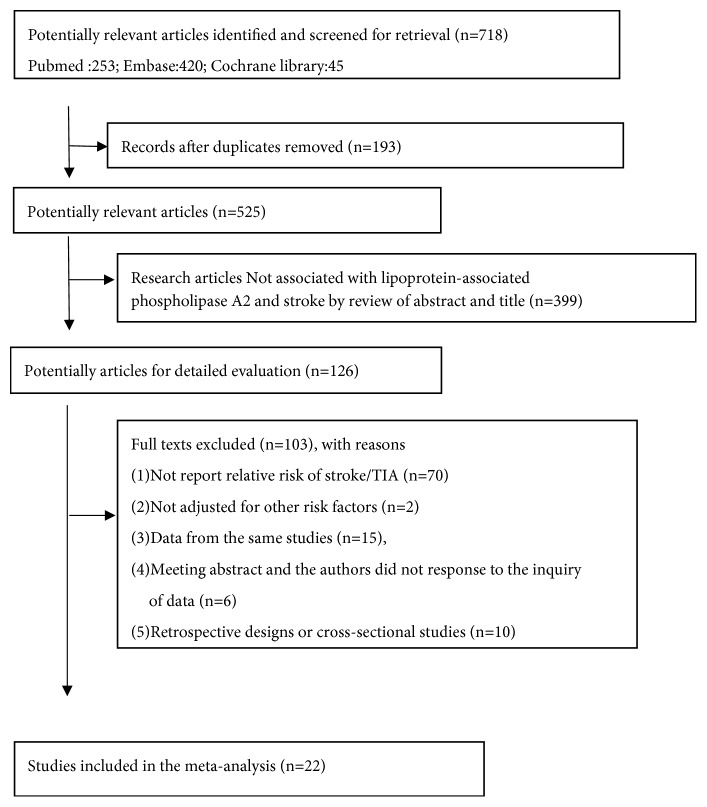
Flow chart of study selection.

From the initial search, 718 publications were identified. After removing 193 duplications, we reviewed titles and abstracts and excluded 399 manuscripts. We then thoroughly assessed the full text from 126 papers. Finally, 22 publications [4, 5, 8–27] were included in our analyses (Figure 1).

The key features of included studies are reported in Table 1; there were 22 studies with 157,693 participants. Among them, the Cardiovascular Health Study (CHS) was separated into two studies by race (CHS-1: whites and CHS-2: African Americans). The Heart Protection Study (HPS) was stratified by stroke subtype (HPS-1: ischemic stroke and HPS-2: hemorrhagic stroke). The results of methodological quality for each included study are presented in Supplement 3. Overall, study quality was of moderate to high quality (ranging from 5–9).

### 3.2. Association of Lp-PLA2 Activity and Stroke Risk

**Figure 2:**
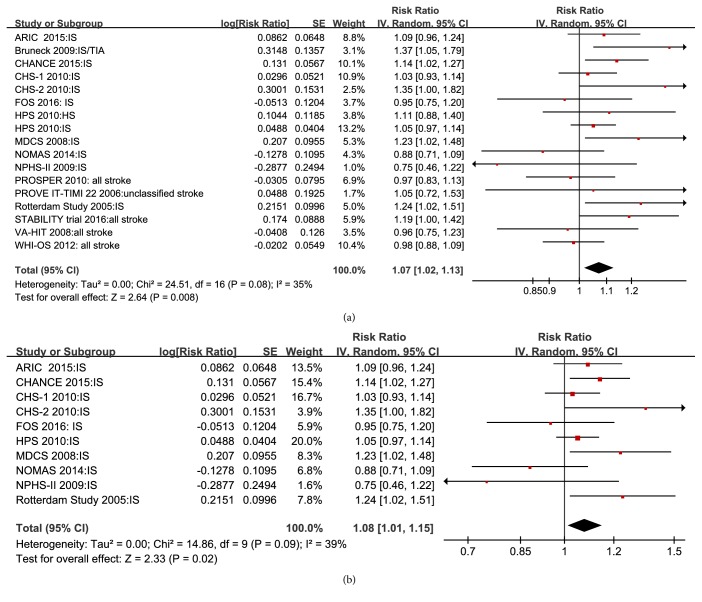
Forest plot of pooled RRs for all stroke (a) and ischemic stroke (b) with 1 SD higher Lp-PLA2 activity.

Seventeen studies reported RR for total stroke with 1 SD higher Lp-PLA2 activity, and the pooled adjusted RR was 1.07 (95% CI 1.02–1.13;* P *= 0.008) in a random-effects model (Figure 2(a)). Similarly, pooled data from nine studies demonstrated that the risk of ischemic stroke with 1 SD higher Lp-PLA2 activity was 1.08 (95% CI 1.01–1.15;* P *= 0.02; Figure 2(b)).

**Figure 3:**
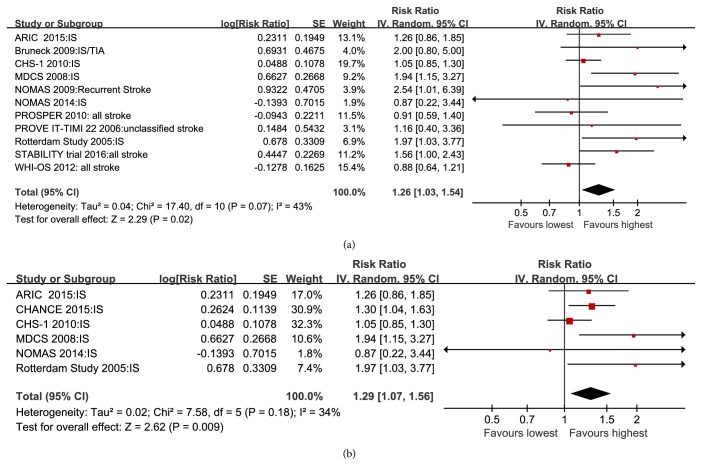
Forest plot of pooled RRs of all stroke (a) and ischemic stroke (b) for Lp-PLA2 activity comparing the highest with the lowest levels of Lp-PLA2.

From 11 studies, the pooled adjusted RR of all stroke when comparing the highest with the lowest Lp-PLA2 activity was 1.26 (95% CI 1.03–1.54;* P* = 0.008) in a random-effects model (Figure 3(a)). Furthermore, the pooled adjusted RR of ischemic stroke comparing the highest with the lowest Lp-PLA2 activity was 1.29 (95% CI 1.07–1.56;* P* = 0.009; Figure 3(b)).

### 3.3. Association of Lp-PLA2 Mass and Stroke Risk

**Figure 4:**
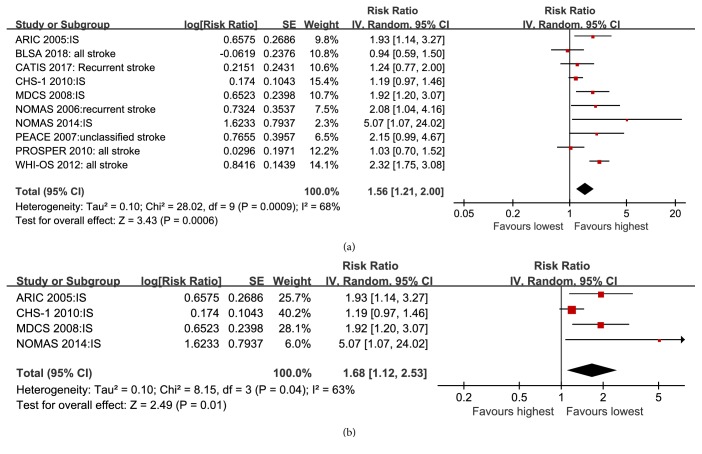
Forest plot of pooled RRs of all stroke (a) and ischemic stroke (b) for Lp-PLA2 mass comparing the highest with the lowest levels of Lp-PLA2.

Data from 10 studies demonstrated that the risk of all stroke when comparing the highest with the lowest Lp-PLA2 mass was 1.56 (95% CI 1.21–2.00;* P *= 0.0006) in a random-effects model (Figure 4(a)). Furthermore, the pooled RR of ischemic stroke comparing the highest with the lowest Lp-PLA2 mass was 1.68 (95% CI 1.12–2.53;* P *= 0.01; Figure 4(b)).

**Figure 5:**
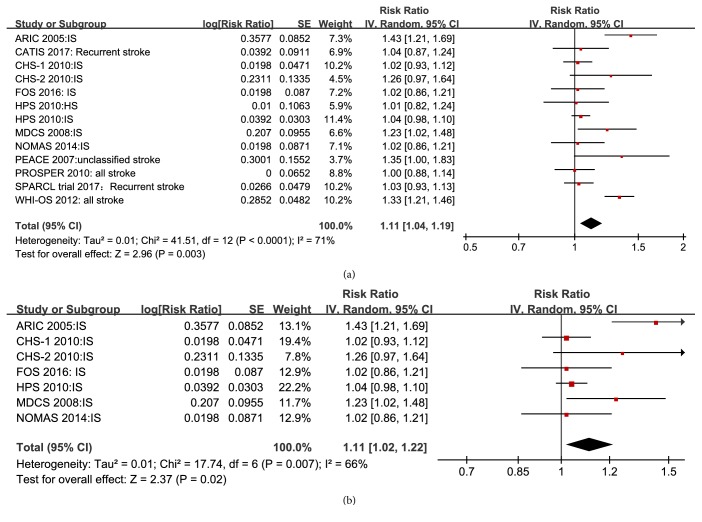
Forest plot of pooled RRs for all stroke (a) and ischemic stroke (b) with 1 SD higher Lp-PLA2 mass.

Thirteen studies reported total stroke risk with 1 SD higher Lp-PLA2 mass. The pooled RR from these studies was 1.11 (95% CI 1.04–1.19;* P* = 0.003; Figure 5(a)). In addition, meta-analysis of seven studies demonstrated that ischemic stroke risk increased per 1 SD increase of Lp-PLA2 mass (RR, 1.11; 95% CI 1.02–1.22;* P *= 0.02; Figure 5(b)).

### 3.4. Publication Bias and Sensitivity Analyses

There was no evidence of publication bias in any analysis, as indicated by Begg's rank correlation test (all* P* > 0.1). Sensitivity analyses demonstrated limited influence in the quantitative pooled RR and its 95% CI for total stroke with 1 SD higher Lp-PLA2 activity and mass. When any individual study was omitted, results remained consistent, suggesting that our conclusion was reliable.

### 3.5. Subgroup Analyses Based on per SD Change of Lp-PLA2 Activity

Elevated Lp-PLA2 activity (per 1 SD) and greater stroke risk were observed consistently in subgroups, except for the subgroups defined as with a prospective cohort study design (RR 1.08; 95% CI 0.99–1.17), mean age ≥ 65 years, follow-up period ≥ 10 years, history of CVD (including coronary heart disease (CHD), stroke and TIA), NOS quality scores < 8 stars, or when men and women were analyzed separately (Supplement 6,7), which may be caused by inadequate numbers and low statistical power. However, none of the differences between these subgroups were significant (all* P* > 0.1).

## 4. Discussion

In this meta-analysis, after adjusting for multiple conventional risk factors, elevated Lp-PLA2 levels of both activity and mass were associated with increased risk of stroke, including ischemic stroke. Per 1 SD higher value of Lp-PLA2 activity and mass, there was 7% and 11% greater risk of all stroke, respectively, and 8% and 11% greater risk of ischemic stroke, respectively. Subjects with the highest Lp-PLA2 activity and mass had respective 26% and 56% greater risk of all stroke compared with those with the lowest activity and mass.

A previous meta-analysis used IPD, and the prognostic strength of Lp-PLA2 for coronary heart disease was determined by a continuous variable analysis [5]. This IPD meta-analysis found that, per 1 SD increase in Lp-PLA 2 mass, there was increased ischemic stroke risk (RR 1.14; 95% CI 1.02–1.27), and per 1 SD increase in LpPLA2 activity, there was significantly increased CHD risk (RR 1.10, 95% CI 1.05–1.16). However, a 1 SD increase in Lp-PLA2 activity was not associated with ischemic stroke risk (RR 1.08; 95% CI 0.97–1.20).

There are two methods to test Lp-PLA2 in human plasma and serum including its plasma concentration (mass) and enzymatic activity. However, Lp-PLA2 mass measurement has been gradually replaced, as it is less accurate than enzymatic activity assessment for risk stratification [28]. The methods for the detection of Lp-PLA2 activity in human blood have been developed and all resulted in more accurate than mass measurement in testing the total levels of circulating Lp-PLA2 [28]. In 2014, The FDA approved a Lp-PLA2 activity test by use of the PLAC® Test (diaDexus Inc, San Francisco, CA, USA) to predict cardiovascular disease in clinical practice.

In our study, elevated Lp-PLA2 activity was related to higher ischemic stroke risk (RR 1.08; 95% CI 1.01–1.15), possibly because we included newer and more relevant studies to increase the statistical power, particularly in the subgroup with mean age < 65 years. There was also an increased trend of all stroke and ischemic stroke risk in subjects with mean age ≥ 65 years with 1-SD-elevated Lp-PLA2 activity levels, although this was not statistically significant. These findings suggest that the relationship between Lp-PLA2 activity levels and ischemic stroke in individuals < 65 years is more pronounced than that in individuals ≥ 65 years. However, this may be because of lack of studies in individuals ≥ 65 years, which leads to low statistical power. Further research into the association between LpPLA2 activity and ischemic stroke in individuals ≥ 65 years is thus needed to confirm this relationship. Patients with 1 SD higher Lp-PLA2 activity had a 16% increased risk of recurrence after cerebral vascular diseases including ischemic stroke, minor stroke, and TIA, demonstrating powerful prognostic strength of Lp-PLA2 activity for stroke in individuals with baseline ischemic stroke, minor stroke, or TIA. However, there was no increased stroke risk in participants with baseline CHD (RR 1.04; 95% CI 0.94–1.15; supplement 7). This may also be because of low statistical power.

The Stroke Council of the American Heart Association/American Stroke Association's updated definition of stroke broadly includes ischemic stroke, hemorrhagic stroke, and cerebral small vessel disease (CSVD, containing white matter hyperintensities (WMH)/silent brain infarcts (SBI) and cerebral microbleeds) [29]. Several of the studies that did not meet the inclusion criteria were cross-sectional studies, reporting relationships between Lp-PLA2 activity and CSVD risk. Performing a pooled RR for CVSD, we found that, per 1 SD increase in Lp-PLA2 activity, there was an increased ischemic CSVD (WMH/SBI) risk (RR 1.31; 95% CI 1.03–1.66; Appendix Supplement 8). Together, these results show that screening of Lp-PLA2 plasma levels may allow for the identification of patients at high risk of future cerebrovascular events, and particularly ischemic cerebrovascular events.

Although high Lp-PLA2 activity and mass were associated with increased risk of CHD and stroke, pharmacological lowering of Lp-PLA2 activity with darapladib does not significantly reduce cardiovascular (including stroke) events in patients who have experienced an ACS or with stable coronary heart disease [11, 30]. Although two trials did not demonstrate clinical efficacy for darapladib, their limitations should be noted. Firstly, clinical efficacy may not be observed over 2.5 years [30] or a median of 3.7 years [11] in these studies. Secondly, the studies did not target a specific degree of Lp-PLA2 inhibition that has efficacy, although darapladib achieved an approximate reduction in Lp-PLA2 of 66% in the SOLID trial [30] and 65% in the STABILITY trial [11]; in addition, many cardiovascular events that occur early after ACS or in stable CHD may be more associated with thrombotic mechanisms and may not be modifiable through Lp-PLA2 inhibition. These reasons may have limited the ability of these studies to discover a difference.

There are limitations to this study. We excluded studies that were unadjusted for confounding factors, which may have introduced bias. There was also a difference among included studies in the definitions of per SD change and highest compared with lowest Lp-PLA2 levels (eg. tertiles, quartiles, quintiles). Finally, the test methods for Lp-PLA2 were not uniform in the included studies, which is a potential source of bias. However, pooled RR in this meta-analysis did not change in the sensitivity analysis, suggesting that the pooled estimate is credible.

In conclusion, elevated Lp-PLA2 levels are associated with higher stroke risk. Lp-PLA2 levels can be considered as factor to predict stroke in high-risk individuals. Lp-PLA2 as a therapeutic target to prevent stroke requires further investigation.

## Figures and Tables

**Table 1:** Characteristics of included studies in the meta-analysis.

Study year: Lead Author	Sample size (% women)	Country/ Region	Age (y), average (SD and/or range)	Study design	Follow-up (y), median or mean (SD and/or range)	Outcome (number)	Quantile of comparison of highest vs. lowest	Reported risk factor (Lp-PLA2 activity and/or mass; Quantile or 1SD)	History of disease (stroke) in baseline	Study quality
ARIC Study 2015: Pokharel	11172 (56)	USA	63(54-74)	Case-cohort	median 11.9	Ischemic Stroke (462)	4 vs. 1 quintile	only activity (both Quintiles and 1SD)	without baseline CHD or ischemic stroke.	9
ARIC Study 2005: Ballantyne	960 (54.8)	USA	57 (45 - 64)	Case-cohort	median 10.6 (1.5-12.7)	Ischemic Stroke (194)	3 vs. 1 tertile	only mass (tertiles)	without baseline CHD or ischemic stroke.	9
BLSA 2018: Wang	1257 (56.2)	China	69.3 (8.1) (>=55)	Cohort	median 5.0	all stroke (113)	Lp-PLA2 < 175 vs >=223ng/mL	only mass(3 categories)	without history of stroke or MI.	9
Bruneck study 2009: Tsimikas	765 (49.6)	Italy	62.7	Cohort	median 10.4 (2.4-10.4)	Ischemic Stroke/TIA (45)	3 vs. 1 tertile	activity (tertiles and reported plot about RR for 1SD activity and IS/TIA)	without baseline TIA or ischemic stroke.	9
CATIS 2017:Han	3401(36.3)	China	62.5 (10.8)	RCT	1	recurrent stroke (162)(all stroke)	4 vs. 1 quartile	mass (quartiles and 1SD)	with acute ischemic stroke	6
CHANCE 2015: Lin	3021 (33.8)	China	62.6 (10.7)	RCT	within a 3-month period	Ischemic Stroke (291)	-	activity (HR for per 30 nmol/min/mL)	with TIA and minor stroke	8
CHS 2010: Jenny	3949 (58.5)	USA	73 (5)	Cohort	mean 12.3 (4.2)	Ischemic Stroke (565)	3 vs. 1 tertile	mass (tertiles) and activity (1SD)	without CVD including MI, stroke and/or CVD death	9
FOS 2016:Shoamanesh	3224(54)	USA	61. 6 (9)	Cohort	mean 9.8 (2.2)	Ischemic Stroke (98)	-	activity and mass (1SD)	Without Stroke or TIA	9
HPS 2010	19037 (25)	UK	64 (8)(40–80)	RCT	mean 5.0	Ischemic (900)+hemorrhagic (96) stroke	-	activity and mass (1SD)	without stroke or (MI) within the previous 6 months	8
MDCS 2008: Persson	5393 (60)	Sweden	58 (6)(46–68)	Cohort	mean 10.6(1.7)	1schemic stroke (152)	3 vs. 1 tertile	activity (tertiles and 1SD); mass (tertiles and 1SD)	without history of MI or stroke.	9
NOMAS 2006: Elkind	467 (54.6)	USA	68.98(12.7)	Cohort	median 4.0	Recurrent stroke (80)	4 vs. 1 quartile	only mass (quartiles)	with ischemic stroke in baseline	7
NOMAS 2009: Elkind	467 (54.6)	USA	68.98(12.7)	Cohort	median 4.0	Recurrent Stroke type unclear (all strokes)(95)	4 vs. 1 quartile	only activity (quartiles)	with ischemic stroke in baseline	7
NOMAS 2014: Katan	1946 (64.4)	USA	69 (10)	Cohort	median 11	1schemic stroke (151)	4 vs. 1 quartile	activity and mass (IS)(1SD); activity and mass (LAA stroke)(quartiles)	stroke-free population	9
NPHS-II 2009: Drenos	2416 (0)	UK	56 (4)	Cohort	median 13.8 (4.7-15.4)	Ischemic Stroke (29)	-	activity (1SD)	free of cardiovascular disease	8
PEACE 2007: Sabatine	3766 (19)	Multinational	64 (8)	RCT	median 4.8	unclassified stroke (87)	4 vs. 1 quartile	mass (quartiles)	with stable CAD	6
PROSPER 2010: Caslake	5804 (52)	UK	aged 70–82	RCT	mean 3.2(2·8–4·0)	all stroke (fatal plus non-fatal stroke)(179)	4 vs. 1 quartile	activity (quartiles and 1SD); mass (quartiles and 1SD))	with stroke or MI or be at high risk of such an event due to a history of hypertension, diabetes or smoking.	6
PROVE IT-TIMI 22 2006: O'Donoghue	3648 (52)	Multinational	58 (11)	RCT	mean 1 (1.5 - 3)	unclassified stroke (30)	5 vs. 1 quintile	only activity (quartiles)	after ACS	6
Rotterdam Study 2005:Oei	7983 (60)	Netherlands	70(>55)	case-cohort study	median 6.4(1.6-8.3)	Ischemic Stroke (110)	4 vs. 1 quartile	activity (1SD and quartiles)	without a history of stroke	9
SPARCL trial 2017: Ganz	2176(38.6)	Multinational	62.9(0.2)	case–cohort	median 5.0	recurrent stroke (562)(all stroke)	-	only mass (1SD)	with Prior Stroke or TIA	7
STABILITY trial 2016:Wallentin L	14500 (18.5)	Multinational	65	RCT	median 3.7	all stroke (280)	4 vs. 1 quartile	only activity (quartiles)	with stable CHD	6
VA-HIT 2008: Robins	1451 (0)	USA	64.1 (7.2)	RCT	mean 5.1	all kinds of stroke (67)	-	only activity (1SD)	with stable CHD	6
WHI-OS 2012: Cook	60890 (100)	USA	63(50 - 79)	prospective case-cohort	median 9.9 (8.6- 11.8)	Ischemic (754)+hemorrhagic (160) stroke	4 vs. 1 quartile	activity (quartiles and 1SD); mass (quartiles and 1SD)	without a history of MI, stroke	8
total	157,693					5,662				

Key: CHD, coronary heart disease; CAD, coronary artery disease; ACS, acute coronary syndrome; MI, myocardial infarction; IS, ischemic stroke; HS, hemorrhagic stroke; TIA, transient ischemic attack; SD, standard deviation; Unclass, unclassified. All kinds of stroke: all stroke included Ischemic Stroke, hemorrhagic stroke and unclassified stroke.

## Data Availability

The detailed data used to support the findings of this study are included within the article and the supplementary material files.

## Supplementary Materials

Supplementary Materials
*Appendix Supplement 1*.PRISMA checklist.* Appendix Supplement 2*.Literature search strategy.* Appendix Supplement 3*.Quality assessment of the included studies.* Appendix Supplement 4*.Confounders adjusted in the included studies.* Appendix Supplement 5*.Assessment of publish bias of included studies by funnel plot and Egger's test in eligible studies.* Appendix Supplement 6*.Table S-1. Subgroup analyses of the association between Lp-PLA2 and risk of stroke.* Appendix Supplement 7*.Subgroup analyses based on per SD change of Lp-PLA2 activity.* Appendix Supplement 8*. Forest plot of Pooled RR and 95%CI for CVSD with 1 SD higher Lp-PLA2 activity.

## References

[1] Donnan G. A., Fisher M., Macleod M., Davis S. M. (2008). Stroke. *The Lancet*.

[2] Asano K., Okamoto S., Fukunaga K. (1999). Cellular source(s) of platelet-activating-factor acetylhydrolase activity in plasma. *Biochemical and Biophysical Research Communications*.

[3] Zalewski A., Macphee C. (2005). Role of lipoprotein-associated phospholipase A2 in atherosclerosis: Biology, epidemiology, and possible therapeutic target. *Arteriosclerosis, Thrombosis, and Vascular Biology*.

[4] O'Donoghue M., Morrow D. A., Sabatine M. S. (2006). Lipoprotein-associated phospholipase A2 and its association with cardiovascular outcomes in patients with acute coronary syndromes in the PROVE IT-TIMI 22 (PRavastatin or atorVastatin evaluation and infection therapy-thrombolysis in myocardial infarction) trial. *Circulation*.

[5] Thompson A., Gao P., Orfei L. (2010). Lipoprotein-associated phospholipase A_2_ and risk of coronary disease, stroke, and mortality: collaborative analysis of 32 prospective studies. *The Lancet*.

[6] Moher D., Liberati A., Tetzlaff J., Altman D. G. (2009). Preferred reporting items for systematic reviews and meta-analyses: the PRISMA statement. *Annals of Internal Medicine*.

[7] Wells G. A., Shea B., O'Connell D. The Newcastle–Ottawa Scale (NOS) for assessing the quality if nonrandomized studies in meta-analyses. http://www.ohri.ca/programs/clinical_epidemiology/oxford.asp.

[8] Wang C., Fang X., Hua Y. (2018). Lipoprotein-associated phospholipase A2 and risk of carotid atherosclerosis and cardiovascular events in community-based older adults in china. *Angiology*.

[9] Han L., Zhong C., Bu X. (2017). Prognostic value of lipoprotein-associated phospholipase A2 mass for all-cause mortality and vascular events within one year after acute ischemic stroke. *Atherosclerosis*.

[10] Ganz P., Amarenco P., Goldstein L. B. (2017). Association of osteopontin, neopterin, and myeloperoxidase with stroke risk in patients with prior stroke or transient ischemic attacks. *Stroke*.

[11] Wallentin L., Held C., Armstrong P. W. (2016). Lipoprotein-associated phospholipase A2 activity is a marker of risk but not a useful target for treatment in patients with stable coronary heart disease. *Journal of the American Heart Association*.

[12] Shoamanesh A., Preis S. R., Beiser A. S. (2016). Circulating biomarkers and incident ischemic stroke in the framingham offspring study. *Neurology*.

[13] Pokharel Y., Sun W., Polfus L. M. (2015). Lipoprotein associated phospholipase A2 activity, apolipoprotein C3 loss-of-function variants and cardiovascular disease: the atherosclerosis risk in communities study. *Atherosclerosis*.

[14] Lin J., Zheng H., Cucchiara B. L. (2015). Association of Lp-PLA 2-A and early recurrence of vascular events after TIA and minor stroke. *Neurology*.

[15] Katan M., Moon Y. P., Paik M. C., Wolfert R. L., Sacco R. L., Elkind M. S. V. (2014). Lipoprotein-associated phospholipase A2 is associated with atherosclerotic stroke risk: the northern manhattan study. *PLoS ONE*.

[16] Cook N. R., Paynter N. P., Manson J. E. (2012). Clinical utility of lipoprotein-associated phospholipase A2 for cardiovascular disease prediction in a multiethnic cohort of women. *Clinical Chemistry*.

[17] Caslake M. J., Packard C. J., Robertson M. (2010). Lipoprotein-associated phospholipase A2, inflammatory biomarkers, and risk of cardiovascular disease in the prospective study of pravastatin in the elderly at risk (PROSPER). *Atherosclerosis*.

[18] Jenny N. S., Solomon C., Cushman M. (2010). Lipoprotein-associated phospholipase A2 (Lp-PLA2) and risk of cardiovascular disease in older adults: results from the cardiovascular health study. *Atherosclerosis*.

[19] HPSGroup (2010). Lipoprotein-associated phospholipase A(2) activity and mass in relation to vascular disease and nonvascular mortality. *Journal of Internal Medicine*.

[20] Tsimikas S., Willeit J., Knoflach M. (2009). Lipoprotein-associated phospholipase A2 activity, ferritin levels, metabolic syndrome, and 10-year cardiovascular and non-cardiovascular mortality: results from the bruneck study. *European Heart Journal*.

[21] Elkind M. S. V., Tai W., Coates K., Paik M. C., Sacco R. L. (2009). Lipoprotein-associated phospholipase A_2_ activity and risk of recurrent stroke. *Cerebrovascular Disease*.

[22] Robins S. J., Collins D., Nelson J. J., Bloomfield H. E., Asztalos B. F. (2008). Cardiovascular events with increased lipoprotein-associated phospholipase A(2) and low high-density lipoprotein-cholesterol: the veterans affairs hdl intervention trial. *Arteriosclerosis, Thrombosis, and Vascular Biology*.

[23] Persson M., Berglund G., Nelson J. J., Hedblad B. (2008). Lp-PLA2 activity and mass are associated with increased incidence of ischemic stroke: a population-based cohort study from Malmo, Sweden. *Atherosclerosis*.

[24] Sabatine M. S., Morrow D. A., O'Donoghue M. (2007). Prognostic utility of lipoprotein-associated phospholipase A2 for cardiovascular outcomes in patients with stable coronary artery disease. *Arteriosclerosis, Thrombosis, and Vascular Biology*.

[25] Elkind M. S. V., Tai W., Coates K., Paik M. C., Sacco R. L. (2006). High-sensitivity C-reactive protein, lipoprotein-associated phospholipase A2, and outcome after ischemic stroke. *JAMA Internal Medicine*.

[26] Ballantyne C. M., Hoogeveen R. C., Bang H. (2005). Lipoprotein-associated phospholipase A2, high-sensitivity C-reactive protein, and risk for incident ischemic stroke in middle-aged men and women in the atherosclerosis risk in communities (ARIC) study. *JAMA Internal Medicine*.

[27] Oei H. H., van der Meer I. M., Hofman A. (2005). Lipoprotein-associated phospholipase A2 activity is associated with risk of coronary heart disease and ischemic stroke: the rotterdam study. *Circulation*.

[28] De Stefano A., Mannucci L., Tamburi F. (2019). Lp-PLA2, a new biomarker of vascular disorders in metabolic diseases. *International Journal of Immunopathology and Pharmacology*.

[29] Sacco R. L., Kasner S. E., Broderick J. P. (2013). An updated definition of stroke for the 21st century: a statement for healthcare professionals from the American heart association/american stroke association. *Stroke*.

[30] O'Donoghue M. L., Braunwald E., White H. D. (2014). Effect of darapladib on major coronary events after an acute coronary syndrome: the SOLID-TIMI 52 randomized clinical trial. *Journal of the American Medical Association*.

