# Supplementary material for: Lipoprotein-Associated Phospholipase A2 Activity and Mass as Independent Risk Factor of Stroke: A Meta-Analysis
Source: Biomed Res Int. 2019 May 20;2019:8642784. doi: 10.1155/2019/8642784 (PMC6545803; doi:10.1155/2019/8642784)
Supplement: Supplementary Materials — Appendix Supplement 1.PRISMA checklist. Appendix Supplement 2.Literature search strategy. Appendix Supplement 3.Quality assessment of the included studies. Appendix Supplement 4.Confounders adjusted in the included studies. Appendix Supplement 5.Assessment of publish bias of included studies by funnel plot and Egger's test in eligible studies. Appendix Supplement 6.Table S-1. Subgroup analyses of the association between Lp-PLA2 and risk of stroke. Appendix Supplement 7.Subgroup analyses based on per SD change of Lp-PLA2 activity. Appendix Supplement 8. Forest plot of Pooled RR and 95%CI for CVSD with 1 SD higher Lp-PLA2 activity. [file 8642784.f1.doc]

**Supplementary Material**

| Appendix Supplement 1 | PRISMA checklist |
| --- | --- |
| Appendix Supplement 2 | Literature search strategy |
| Appendix Supplement 3 | Quality assessment of the included studies |
| Appendix Supplement 4 | Confounders adjusted in the included studies |
| Appendix Supplement 5 | Assessment of publish bias of included studies by funnel plot and Egger’s test in eligible studies |
| Appendix Supplement 6 | Table S-1. Subgroup analyses of the association between Lp-PLA2 and risk of stroke |
| Appendix Supplement 7 | Subgroup analyses based on per SD change of Lp-PLA2 activity |
| Appendix Supplement 8 | Forest plot of Pooled RR and 95%CI for CVSD with 1 SD higher Lp-PLA2 activity |

**Appendix Supplement 1**. PRISMA checklist

| Section/topic | Item No | Checklist item | Reported on page No |
| --- | --- | --- | --- |
| Title | | | |
| Title | 1 | Identify the report as a systematic review, meta-analysis, or both | 1 |
| Abstract | | | |
| Structured summary | 2 | Provide a structured summary including, as applicable, background, objectives, data sources, study eligibility criteria, participants, interventions, study appraisal and synthesis methods, results, limitations, conclusions and implications of key findings | 2-3 |
| Introduction | | | |
| Rationale | 3 | Describe the rationale for the review in the context of what is already known | 4-5 |
| Objectives | 4 | Provide an explicit statement of questions being addressed with reference to participants, interventions, comparisons, outcomes, and study design (PICOS) | 4-5 |
| Methods | | | |
| Protocol and registration | 5 | Indicate if a review protocol exists, if and where it can be accessed (such as web address), and, if available, provide registration information including registration number | 5 |
| Eligibility criteria | 6 | Specify study characteristics (such as PICOS, length of follow-up) and report characteristics (such as years considered, language, publication status) used as criteria for eligibility, giving rationale | 6 |
| Information sources | 7 | Describe all information sources (such as databases with dates of coverage, contact with study authors to identify additional studies) in the search and date last searched | 6 |
| Search | 8 | Present full electronic search strategy for at least one database, including any limits used, such that it could be repeated | 5 and Supplement 2 |
| Study selection | 9 | State the process for selecting studies (that is, screening, eligibility, included in systematic review, and, if applicable, included in the meta-analysis) | 6 |
| Data collection process | 10 | Describe method of data extraction from reports (such as piloted forms, independently, in duplicate) and any processes for obtaining and confirming data from investigators | 6 |
| Data items | 11 | List and define all variables for which data were sought (such as PICOS, funding sources) and any assumptions and simplifications made | 6(table 1) |
| Risk of bias in individual studies | 12 | Describe methods used for assessing risk of bias of individual studies (including specification of whether this was done at the study or outcome level), and how this information is to be used in any data synthesis | 6 |
| Summary measures | 13 | State the principal summary measures (such as risk ratio, difference in means). | 7 |
| Synthesis of results | 14 | Describe the methods of handling data and combining results of studies, if done, including measures of consistency (such as I2 statistic) for each meta-analysis | 7 |
| Risk of bias across studies | 15 | Specify any assessment of risk of bias that may affect the cumulative evidence (such as publication bias, selective reporting within studies) | 7 |
| Additional analyses | 16 | Describe methods of additional analyses (such as sensitivity or subgroup analyses, meta-regression), if done, indicating which were pre-specified | 7 |
| Results | | | |
| Study selection | 17 | Give numbers of studies screened, assessed for eligibility, and included in the review, with reasons for exclusions at each stage, ideally with a flow diagram | 8 and Figure 1 |
| Study characteristics | 18 | For each study, present characteristics for which data were extracted (such as study size, PICOS, follow-up period) and provide the citations | 8, Table 1 |
| Risk of bias within studies | 19 | Present data on risk of bias of each study and, if available, any outcome-level assessment (see item 12). | 8, Appendix Supplement 3 |
| Results of individual studies | 20 | For all outcomes considered (benefits or harms), present for each study (a) simple summary data for each intervention group and (b) effect estimates and confidence intervals, ideally with a forest plot | 9 |
| Synthesis of results | 21 | Present results of each meta-analysis done, including confidence intervals and measures of consistency | 9 |
| Risk of bias across studies | 22 | Present results of any assessment of risk of bias across studies (see item 15) | 10 |
| Additional analysis | 23 | Give results of additional analyses, if done (such as sensitivity or subgroup analyses, meta-regression) (see item 16) | 10 |
| Discussion | | | |
| Summary of evidence | 24 | Summarise the main findings including the strength of evidence for each main outcome; consider their relevance to key groups (such as health care providers, users, and policy makers) | 11-14 |
| Limitations | 25 | Discuss limitations at study and outcome level (such as risk of bias), and at review level (such as incomplete retrieval of identified research, reporting bias) | 14 |
| Conclusions | 26 | Provide a general interpretation of the results in the context of other evidence, and implications for future research | 14 |
| Funding | | | |
| Funding | 27 | Describe sources of funding for the systematic review and other support (such as supply of data) and role of funders for the systematic review | 15 |

**Appendix Supplement 2.** Literature search strategy for all the databases

1. Search strategy for Pubmed

# 1 1-Alkyl-2-acetylglycerophosphocholine Esterase [Mesh]

#2 (1 Alkyl 2 acetylglycerophosphocholine Esterase) or (Esterase, 1-Alkyl-2-acetylglycerophosphocholine) or (PAF Acetylhydrolase II) or (Acetylhydrolase II, PAF) or (Platelet-Activating Factor Hydrolase) or (Factor Hydrolase, Platelet-Activating) or (Hydrolase, Platelet-Activating Factor) or (Platelet Activating Factor Hydrolase) or (PAF 2-Acylhydrolase) or (PAF 2 Acylhydrolase) or (Lipoprotein-Associated Phospholipase A2) or (Lipoprotein Associated Phospholipase A2) or (Phospholipase A2, Lipoprotein-Associated) or (Lp-PLA(2)) or (Lp-PLA2) or (Lp PLA2) or (PAF 2-Acetylhydrolase) or (2-Acetylhydrolase, PAF) or (PAF 2 Acetylhydrolase) or (PAF Acetylhydrolase) or (Acetylhydrolase, PAF) or (Lipoprotein-Associated Phospholipase A(2)) or (Platelet-activating Factor Acetylhydrolase IB) or (Platelet activating Factor Acetylhydrolase IB)

# 3 # 1 or # 2

# 4 stroke [Mesh]

# 5 (CVA (Cerebrovascular Accident)) or (CVAs (Cerebrovascular Accident)) or (Cerebrovascular Accident) or (Cerebrovascular Accidents) or (Cerebrovascular Apoplexy) or (Apoplexy, Cerebrovascular) or (Cerebrovascular Stroke) or (Cerebrovascular Strokes) or (Stroke, Cerebrovascular) or (Strokes, Cerebrovascular) or (Vascular Accident, Brain) or (Brain Vascular Accident) or (Brain Vascular Accidents) or (Vascular Accidents, Brain) or (Cerebral Stroke) or (Cerebral Strokes) or (Stroke, Cerebral) or (Strokes, Cerebral) or (Stroke, Acute) or (Acute Stroke) or (Acute Strokes) or (Strokes, Acute) or (Cerebrovascular Accident, Acute) or (Acute Cerebrovascular Accident) or (Acute Cerebrovascular Accidents) or (Cerebrovascular Accidents, Acute) or (cerebrovascular disease) or (cerebrovascular attack) or (cerebral ischemia) or (brain ischemia) or (cerebrovascular disorders) or (basal ganglia cerebrovascular disease) or (brain ischemia) or (brain infarction) or (hypoxia-ischemia,brain) or (carotid artery diseases) or (carotid artery thrombosis) or (carotid artery, internal, dissection) or (intracranial arterial diseases) or (cerebral arterial diseases) or (infarction, anterior cerebral artery) or (infarction, middle cerebral artery) or (infarction, posterior cerebral artery) or (intracranial embolism and thrombosis) or (vertebral artery dissection)

# 6 ((ischemi* or ischaemi*) AND ((stroke* or apoplex* or cerebral vasc* or cerebrovasc* or cva or attack*)))

# 7 ((brain or cerebr* or cerebell* or vertebrobasil* or hemisphere* or intracran* or intracerebral or infratentorial or supratentorial or middle cerebr* or mca* or anterior circulation) and (ischemi* or infarct* or thrombo* or emboli* or occlus* or hypoxi*))

# 8 # 4 or # 5 or # 6 or # 7

# 9 "Intracranial Hemorrhages"[Mesh] or "Intracranial Hemorrhage, Hypertensive"[Mesh] or "Subarachnoid Hemorrhage"[Mesh]

# 10 (Hemorrhages, Intracranial) or (Intracranial Hemorrhage) or (Hemorrhage, Intracranial) or (Posterior Fossa Hemorrhage) or (Hemorrhage, Posterior Fossa) or (Hemorrhages, Posterior Fossa) or (Posterior Fossa Hemorrhages) or (Brain Hemorrhage) or (Brain Hemorrhages) or (Hemorrhage, Brain) or (Hemorrhages, Brain)

# 11 [(hemorrhage, hypertensive intracranial) or (hemorrhages, hypertensive intracranial) or (hypertensive intracranial hemorrhage) or (hypertensive intracranial hemorrhages) or (intracranial hemorrhages, hypertensive) or (hypertensive hemorrhage, intracranial) or (hemorrhage, intracranial hypertensive) or (hemorrhages, intracranial hypertensive) or (hypertensive hemorrhages, intracranial) or (intracranial hypertension hemorrhage) or (intracranial hypertension hemorrhages) or (cerebral hemorrhage, hypertensive) or (cerebral hemorrhages, hypertensive) or (hemorrhage, hypertensive cerebral) or (hemorrhages, hypertensive cerebral) or (hypertensive cerebral hemorrhage) or (hypertensive cerebral hemorrhages) or (intracerebral hemorrhage, hypertensive) or (hemorrhage, hypertensive intracerebral) or (hemorrhages, hypertensive intracerebral) or (hypertensive intracerebral hemorrhage) or (hypertensive intracerebral hemorrhages) or (intracerebral hemorrhages, hypertensive) or (hypertensive hemorrhage, cerebral) or (cerebral hypertensive hemorrhage) or (cerebral hypertensive hemorrhage) or (hemorrhage, cerebral hypertensive) or (hemorrhages, cerebral hypertensive) or (hypertensive hemorrhages, cerebral)](http://www.ncbi.nlm.nih.gov/pubmed?term=(hemorrhage, hypertensive intracranial) OR (hemorrhages, hypertensive intracranial) OR (hypertensive intracranial hemorrhage) OR (hypertensive intracranial hemorrhages) OR (intracranial hemorrhages, hypertensive) OR (hypertensive hemorrhage, intracranial) OR (hemorrhage, intracranial hypertensive) OR (hemorrhages, intracranial hypertensive) OR (hypertensive hemorrhages, intracranial) OR (intracranial hypertension hemorrhage) OR (intracranial hypertension hemorrhages) OR (cerebral hemorrhage, hypertensive) OR (cerebral hemorrhages, hypertensive) OR (hemorrhage, hypertensive cerebral) OR (hemorrhages, hypertensive cerebral) OR (hypertensive cerebral hemorrhage) OR (hypertensive cerebral hemorrhages) OR (intracerebral hemorrhage, hypertensive) OR (hemorrhage, hypertensive intracerebral) OR (hemorrhages, hypertensive intracerebral) OR (hypertensive intracerebral hemorrhage) OR (hypertensive intracerebral hemorrhages) OR (intracerebral hemorrhages, hypertensive) OR (hypertensive hemorrhage, cerebral) OR (cerebral hypertensive hemorrhage) OR (cerebral hypertensive hemorrhage) OR (hemorrhage, cerebral hypertensive) OR (hemorrhages, cerebral hypertensive) OR (hypertensive hemorrhages, cerebral)&cmd=correctspelling)

# 12 (SAH (Subarachnoid Hemorrhage)) or (SAHs (Subarachnoid Hemorrhage)) or (Hemorrhage, Subarachnoid) or (Hemorrhages, Subarachnoid) or (Subarachnoid Hemorrhages) or (Subarachnoid Hemorrhage, Aneurysmal) or (Aneurysmal Subarachnoid Hemorrhage) or (Aneurysmal Subarachnoid Hemorrhages) or (Hemorrhage, Aneurysmal Subarachnoid) or (Hemorrhages, Aneurysmal Subarachnoid) or (Subarachnoid Hemorrhages, Aneurysmal) or (Subarachnoid Hemorrhage, Spontaneous) or (Hemorrhage, Spontaneous Subarachnoid) or (Hemorrhages, Spontaneous Subarachnoid) or (Spontaneous Subarachnoid Hemorrhage) or (Spontaneous Subarachnoid Hemorrhages) or (Subarachnoid Hemorrhages, Spontaneous) or (Perinatal Subarachnoid Hemorrhage) or (Hemorrhage, Perinatal Subarachnoid) or (Hemorrhages, Perinatal Subarachnoid) or (Perinatal Subarachnoid Hemorrhages) or (Subarachnoid Hemorrhage, Perinatal) or (Subarachnoid Hemorrhages, Perinatal) or (Subarachnoid Hemorrhage, Intracranial) or (Hemorrhage, Intracranial Subarachnoid) or (Hemorrhages, Intracranial Subarachnoid) or (Intracranial Subarachnoid Hemorrhage) or (Intracranial Subarachnoid Hemorrhages) or (Subarachnoid Hemorrhages, Intracranial)

# 13 #9 or # 10 or # 11 or # 12

# 14 #8 or # 13

# 15 "Ischemic Attack, Transient"[Mesh]

#16. (TIA (Transient Ischemic Attack)) or (TIAs (Transient Ischemic Attack)) or (Transient Ischemic Attack) or (Attack, Transient Ischemic) or (Attacks, Transient Ischemic) or (Ischemic Attacks, Transient) or (Transient Ischemic Attacks) or (Brain TIA) or (TIA, Brain) or (Carotid Circulation Transient Ischemic Attack) or (Transient Ischemic Attack, Carotid Circulation) or (Transient Ischemic Attack, Vertebrobasilar Circulation) or (Vertebrobasilar Circulation Transient Ischemic Attack) or (Crescendo Transient Ischemic Attacks) or (Transient Ischemic Attacks, Crescendo) or (Posterior Circulation Transient Ischemic Attack) or (Transient Ischemic Attack, Posterior Circulation) or (Transient Ischemic Attack, Anterior Circulation) or (Anterior Circulation Transient Ischemic Attack) or (Brain Stem Ischemia, Transient) or (Transient Ischemic Attack, Brain Stem) or (Brainstem Ischemia, Transient) or (Brainstem Ischemias, Transient) or (Ischemia, Transient Brainstem) or (Ischemias, Transient Brainstem) or (Transient Brainstem Ischemia) or (Brainstem Transient Ischemic Attack) or (Transient Ischemic Attack, Brainstem) or (Brain Stem Transient Ischemic Attack) or (Cerebral Ischemia, Transient) or (Cerebral Ischemias, Transient) or (Ischemia, Transient Cerebral) or (Ischemias, Transient Cerebral) or (Transient Cerebral Ischemia) or (Transient Cerebral Ischemias)

# 17 # 15 or # 16

# 18 # 14 or # 17

# 19 # 3 AND # 18

2. Search strategy for Embase

#1 '1 alkyl 2 acetylglycerophosphocholine esterase'/exp

#2. ‘1 Alkyl 2 acetylglycerophosphocholine Esterase’ or ‘PAF Acetylhydrolase II’ or ‘Platelet-Activating Factor Hydrolase’ or ‘Platelet Activating Factor Hydrolase’ or ‘PAF 2-Acylhydrolase’ or ‘PAF 2 Acylhydrolase’ or ‘Lipoprotein-Associated Phospholipase A2’ or ‘Lipoprotein Associated Phospholipase A2’ or ‘Lp-PLA2’ or ‘Lp-PLA2’ or ‘Lp PLA2’ or ‘PAF 2-Acetylhydrolase’ or ‘PAF 2 Acetylhydrolase’ or ‘PAF Acetylhydrolase’ or ‘Lipoprotein-Associated Phospholipase A(2)’ or ‘Platelet-activating Factor Acetylhydrolase IB’ or ‘Platelet activating Factor Acetylhydrolase IB’

#3 #1 or #2

#4 'cerebrovascular accident'/exp or ‘brain ischemia’/exp or ‘carotid artery obstruction’/exp or ‘occlusive cerebrovascular disease’/exp

#5 ‘cerebrovascular disease’ or 'cerebrovascular accident' or ‘brain infarction’ or ‘brain stem infarction’ or ‘cerebellum infarction’ or ‘carotid artery disease’ or ‘cerebral artery disease’ or ‘stroke patient’ .tw

#6 ‘ischaemic stroke’ or ‘ischemic stroke’ .tw
#7 ‘haemorrhagic stroke’.tw

#8 ‘intracerebral haemorrhage’.tw

#9 stroke.tw

#10 (stroke$ or poststroke$ or cva$).tw

#11 (cerebrovascular$ or cerebral vascular)

#12(cerebral or cerebellar or brainstem or vertebrobasilar or brain) and (Infarct$ or isch?emi$ or thrombo$ or apoplexy or emboli$)

# 13 (cerebral or intracerebral or intracranial or brain or brainstem or cerebellar or vertebrobasilar) and (haemorrhage$ or hemorrhag$ or haematoma or hematoma or bleed$)

#14 #4 or #5 or #6 or #7 or #8 or #9 or #10 or #11 or #12 or #13

#15 #3 AND #14

3. Search strategy for Cochrane

#1 MeSH descriptor: [1-Alkyl-2-acetylglycerophosphocholine Esterase] explode all trees

#2 (1 Alkyl 2 acetylglycerophosphocholine Esterase) or (Esterase, 1 Alkyl 2 acetylglycerophosphocholine) or (PAF Acetylhydrolase II) or (Acetylhydrolase II, PAF) or (Platelet Activating Factor Hydrolase) or (Factor Hydrolase, Platelet Activating) or (Hydrolase, Platelet Activating Factor) or (Platelet Activating Factor Hydrolase) or (PAF 2 Acylhydrolase) or (PAF 2 Acylhydrolase) or (Lipoprotein Associated Phospholipase A2) or (Lipoprotein Associated Phospholipase A2) or (Phospholipase A2, Lipoprotein Associated) or (Lp PLA (2)) or (Lp PLA2) or (Lp PLA2) or (PAF 2 Acetylhydrolase) or (2 Acetylhydrolase, PAF) or (PAF 2 Acetylhydrolase) or (PAF Acetylhydrolase) or (Acetylhydrolase, PAF) or (Lipoprotein Associated Phospholipase A (2)) or (Platelet activating Factor Acetylhydrolase IB) or (Platelet activating Factor Acetylhydrolase IB):ti,ab,kw (Word variations have been searched) 172

#3 #1 or #2 172

#4 MeSH descriptor: [Stroke] explode all trees 6571

#5 (CVA (Cerebrovascular Accident)) or (CVAs (Cerebrovascular Accident)) or (Cerebrovascular Accident) or (Cerebrovascular Accidents) or (Cerebrovascular Apoplexy) or (Apoplexy, Cerebrovascular) or (Cerebrovascular Stroke) or (Cerebrovascular Strokes) or (Stroke, Cerebrovascular) or (Strokes, Cerebrovascular) or (Vascular Accident, Brain) or (Brain Vascular Accident) or (Brain Vascular Accidents) or (Vascular Accidents, Brain) or (Cerebral Stroke) or (Cerebral Strokes) or (Stroke, Cerebral) or (Strokes, Cerebral) or (Stroke, Acute) or (Acute Stroke) or (Acute Strokes) or (Strokes, Acute) or (Cerebrovascular Accident, Acute) or (Acute Cerebrovascular Accident) or (Acute Cerebrovascular Accidents) or (Cerebrovascular Accidents, Acute) or (cerebrovascular disease) or (cerebrovascular attack) or (cerebral ischemia) or (brain ischemia) or (cerebrovascular disorders) or (basal ganglia cerebrovascular disease) or (brain ischemia) or (brain infarction) or (hypoxia-ischemia,brain) or (carotid artery diseases) or (carotid artery thrombosis) or (carotid artery, internal, dissection) or (intracranial arterial diseases) or (cerebral arterial diseases) or (infarction, anterior cerebral artery) or (infarction, middle cerebral artery) or (infarction, posterior cerebral artery) or (intracranial embolism and thrombosis) or (vertebral artery dissection):ti,ab,kw (Word variations have been searched) 18467

#6 ((ischemi* or ischaemi*) and (stroke* or apoplex* or cerebral vasc* or cerebrovasc* or cva or attack*)):ti,ab,kw (Word variations have been searched) 9317

#7 ((brain or cerebr* or cerebell* or vertebrobasil* or hemisphere* or intracran* or intracerebral or infratentorial or supratentorial or middle cerebr* or mca* or anterior circulation) and (ischemi* or infarct* or thrombo* or emboli* or occlus* or hypoxi*)):ti,ab,kw (Word variations have been searched) 12213

#8 #4 or #5 or #6 or #7 28082

#9 MeSH descriptor: [Intracranial Hemorrhages] explode all trees 1512

#10 MeSH descriptor: [Intracranial Hemorrhage, Hypertensive] explode all trees 17

#11 MeSH descriptor: [Subarachnoid Hemorrhage] explode all trees 494

#12 #9 or #10 or #11 1512

#13 (Hemorrhages, Intracranial) or (Intracranial Hemorrhage) or (Hemorrhage, Intracranial) or (Posterior Fossa Hemorrhage) or (Hemorrhage, Posterior Fossa) or (Hemorrhages, Posterior Fossa) or (Posterior Fossa Hemorrhages) or (Brain Hemorrhage) or (Brain Hemorrhages) or (Hemorrhage, Brain) or (Hemorrhages, Brain):ti,ab,kw (Word variations have been searched) 3234

#14 (hemorrhage, hypertensive intracranial) or (hemorrhages, hypertensive intracranial) or (hypertensive intracranial hemorrhage) or (hypertensive intracranial hemorrhages) or (intracranial hemorrhages, hypertensive) or (hypertensive hemorrhage, intracranial) or (hemorrhage, intracranial hypertensive) or (hemorrhages, intracranial hypertensive) or (hypertensive hemorrhages, intracranial) or (intracranial hypertension hemorrhage) or (intracranial hypertension hemorrhages) or (cerebral hemorrhage, hypertensive) or (cerebral hemorrhages, hypertensive) or (hemorrhage, hypertensive cerebral) or (hemorrhages, hypertensive cerebral) or (hypertensive cerebral hemorrhage) or (hypertensive cerebral hemorrhages) or (intracerebral hemorrhage, hypertensive) or (hemorrhage, hypertensive intracerebral) or (hemorrhages, hypertensive intracerebral) or (hypertensive intracerebral hemorrhage) or (hypertensive intracerebral hemorrhages) or (intracerebral hemorrhages, hypertensive) or (hypertensive hemorrhage, cerebral) or (cerebral hypertensive hemorrhage) or (cerebral hypertensive hemorrhage) or (hemorrhage, cerebral hypertensive) or (hemorrhages, cerebral hypertensive) or (hypertensive hemorrhages, cerebral):ti,ab,kw (Word variations have been searched) 260

#15 (SAH (Subarachnoid Hemorrhage)) or (SAHs (Subarachnoid Hemorrhage)) or (Hemorrhage, Subarachnoid) or (Hemorrhages, Subarachnoid) or (Subarachnoid Hemorrhages) or (Subarachnoid Hemorrhage, Aneurysmal) or (Aneurysmal Subarachnoid Hemorrhage) or (Aneurysmal Subarachnoid Hemorrhages) or (Hemorrhage, Aneurysmal Subarachnoid) or (Hemorrhages, Aneurysmal Subarachnoid) or (Subarachnoid Hemorrhages, Aneurysmal) or (Subarachnoid Hemorrhage, Spontaneous) or (Hemorrhage, Spontaneous Subarachnoid) or (Hemorrhages, Spontaneous Subarachnoid) or (Spontaneous Subarachnoid Hemorrhage) or (Spontaneous Subarachnoid Hemorrhages) or (Subarachnoid Hemorrhages, Spontaneous) or (Perinatal Subarachnoid Hemorrhage) or (Hemorrhage, Perinatal Subarachnoid) or (Hemorrhages, Perinatal Subarachnoid) or (Perinatal Subarachnoid Hemorrhages) or (Subarachnoid Hemorrhage, Perinatal) or (Subarachnoid Hemorrhages, Perinatal) or (Subarachnoid Hemorrhage, Intracranial) or (Hemorrhage, Intracranial Subarachnoid) or (Hemorrhages, Intracranial Subarachnoid) or (Intracranial Subarachnoid Hemorrhage) or (Intracranial Subarachnoid Hemorrhages) or (Subarachnoid Hemorrhages, Intracranial):ti,ab,kw 1283

#16 #13 or #14 or #15 3905

#17 #8 or #12 or #16 29835

#18 MeSH descriptor: [Ischemic Attack, Transient] explode all trees 614

#19 (TIA (Transient Ischemic Attack)) or (TIAs (Transient Ischemic Attack)) or (Transient Ischemic Attack) or (Attack, Transient Ischemic) or (Attacks, Transient Ischemic) or (Ischemic Attacks, Transient) or (Transient Ischemic Attacks) or (Brain TIA) or (TIA, Brain) or (Carotid Circulation Transient Ischemic Attack) or (Transient Ischemic Attack, Carotid Circulation) or (Transient Ischemic Attack, Vertebrobasilar Circulation) or (Vertebrobasilar Circulation Transient Ischemic Attack) or (Crescendo Transient Ischemic Attacks) or (Transient Ischemic Attacks, Crescendo) or (Posterior Circulation Transient Ischemic Attack) or (Transient Ischemic Attack, Posterior Circulation) or (Transient Ischemic Attack, Anterior Circulation) or (Anterior Circulation Transient Ischemic Attack) or (Brain Stem Ischemia, Transient) or (Transient Ischemic Attack, Brain Stem) or (Brainstem Ischemia, Transient) or (Brainstem Ischemias, Transient) or (Ischemia, Transient Brainstem) or (Ischemias, Transient Brainstem) or (Transient Brainstem Ischemia) or (Brainstem Transient Ischemic Attack) or (Transient Ischemic Attack, Brainstem) or (Brain Stem Transient Ischemic Attack) or (Cerebral Ischemia, Transient) or (Cerebral Ischemias, Transient) or (Ischemia, Transient Cerebral) or (Ischemias, Transient Cerebral) or (Transient Cerebral Ischemia) or (Transient Cerebral Ischemias) :ti,ab,kw 1961

#20 #18 or #19 1961

#21 #17 or #20 29940

#22 #3 and #21 22

**Appendix Supplement 3.** Quality assessment of the included studies in meta-analysis

| Study | Rrepresentativeness of the exposed cohort | Selection of the non exposed cohort | Ascertainment of exposure | Demonstration that outcome was not present at study start | Comparability of cohorts on the basis of the design or analysis | Enough Assessment of outcome | Enough follow-up periods for outcome to occur (≥2 years or outcome of all patients occurred ) | Documented rate of loss to follow-up<=25% (or no loss to follow-up) | Overall NOS scores |
| --- | --- | --- | --- | --- | --- | --- | --- | --- | --- |
| ARIC Study 2015 | ★ | ★ | ★ | ★ | ★★ | ★ | ★ | ★ | 9 |
| ARIC Study 2005 | ★ | ★ | ★ | ★ | ★★ | ★ | ★ | ★ | 9 |
| BLSA 2018 | ★ | ★ | ★ | ★ | ★★ | ★ | ★ | ★ | 9 |
| Bruneck study 2009 | ★ | ★ | ★ | ★ | ★★ | ★ | ★ | ★ | 9 |
| CATIS 2017 |  | ★ | ★ |  | ★★ | ★ |  | ★ | 6 |
| CHANCE 2015 | ★ | ★ | ★ |  | ★★ | ★ | ★ | ★ | 8 |
| CHS 2010 | ★ | ★ | ★ | ★ | ★★ | ★ | ★ | ★ | 9 |
| FOS 2016 | ★ | ★ | ★ | ★ | ★★ | ★ | ★ | ★ | 9 |
| HPS 2010 |  | ★ | ★ | ★ | ★★ | ★ | ★ | ★ | 8 |
| MDCS 2008 | ★ | ★ | ★ | ★ | ★★ | ★ | ★ | ★ | 9 |
| NOMAS 2014 | ★ | ★ | ★ | ★ | ★★ | ★ | ★ | ★ | 9 |
| NOMAS 2006 2009 |  | ★ | ★ |  | ★★ | ★ | ★ | ★ | 7 |
| NPHS-II 2009 | ★ | ★ | ★ | ★ | ★★ | ★ | ★ |  | 8 |
| PEACE 2007 |  | ★ | ★ |  | ★★ | ★ | ★ |  | 6 |
| PROVEIT-TIMI22 2006 |  | ★ | ★ |  | ★★ | ★ | ★ |  | 6 |
| PROSPER 2010 |  | ★ | ★ |  | ★★ | ★ | ★ |  | 6 |
| Rotterdam 2005 | ★ | ★ | ★ | ★ | ★★ | ★ | ★ | ★ | 9 |
| SPARCL trial 2017 |  | ★ | ★ |  | ★★ | ★ | ★ | ★ | 7 |
| STABILITY trial 2016 |  | ★ | ★ |  | ★★ | ★ | ★ |  | 6 |
| VA-HIT 2008 |  | ★ | ★ |  | ★★ | ★ | ★ |  | 6 |
| WHI-OS 2012 | ★ | ★ | ★ | ★ | ★★ | ★ | ★ |  | 8 |

Note: The NOS assigns one point for comparability when RRs were adjusted for age and/or sex; and assigns two points for comparability when further adjustment for other risk factors.

**Appendix Supplement 4.** Confounders adjusted in the included studies

| Study | Risk factors adjusted |
| --- | --- |
| ARIC Study 2015 | age, gender, and race, current smoking, systolic blood pressure, antihypertensive medication use, diabetes, log hs-CRP，HDL-C , LDL-C. |
| ARIC Study 2005 | age, sex, race, smoking status, systolic blood pressure, LDL-C and HDL-C levels, diabetes, hs-CRP level, antihypertensive medication, and body mass index. |
| BLSA 2018 | age, gender, smoking, uric acid, diabetes, pulse pressure, systolic blood pressure, hsCRP, dyslipidemia, CCA-IMT, and carotid plaque presence. |
| Bruneck study 2009 | age, sex, previous cardiovascular disease, systolic blood pressure, smoking, diabetes, ferritin level, fibrinogen level, LDL and HDL cholesterol, waist-to-hip ratio, alcohol consumption, social status, sports activity, and loge-transformed levels of HOMA-IR, lipoprotein(a), C-reactive protein, and urinary albumin. |
| CATIS 2017 | age, sex, body mass index，admission NIHSS score, time from onset to randomization, current smoking, alcohol drinking, systolic blood pressure, plasma glucose,triglyceride, total cholesterol, HDL-cholesterol, estimated glomerular filtration rate, high sensitivity C-reactive protein, history of hypertension, history of hyperlipidemia, history of coronary heart disease, history of diabetes, ischemic stroke subtype, use of antihypertensive and lipid-lowering medications, and randomized treatment，LDL-C level. |
| CHANCE 2015 | age, sex, BMI, systolic blood pressure, History of hypertension, History of diabetes, History of myocardial infarction, and anti-platelet treatment |
| CHS 2010 | age, sex, ethnicity，BMI, diabetes, smoking, hypertension, dyslipidemia，CRP (by cutpoints). |
| FOS 2016 | age at examination 7, sex, systolic blood pressure, hypertension treatment, current smoking, history of diabetes, history of cardiovascular disease, history of atrial fibrillation. |
| HPS 2010 | simvastatin allocation, age, sex, prior vascular disease, systolic blood pressure, smoking, estimated glomerular filtration rate and medication (‘basic covariates’), the weighted average of screening and randomization values of apoA1 and apo B. |
| MDCS 2008 | age, sex, LDL, HDL, lipid lowering treatment, BMI, systolic blood pressure, blood pressure lowering treatment, diabetes mellitus, smoking, hsCRP and alcohol consumption |
| NOMAS 2009 | age, sex, race/ethnicity, history of hypertension, diabetes mellitus, hyperlipidemia, smoking, coronary artery disease, hsCRP, LDL, and the interaction with LDL ≥130 mg/dl. |
| NOMAS 2014 | age, sex, race-ethnicity, education, waist circumference, physical activity, moderate alcohol consumption, smoker, diabetes mellitus, systolic blood pressure, coronary artery disease, LDL, HDL |
| NPHS-II 2009 | age and history of diabetes，lipid-lowering drug use，systolic blood pressure，body-mass index，smoking status， non-HDL cholesterol，HDL cholesterol，loge triglyceride（data from lancet IPD） |
| PEACE 2007 | age, sex, race, hypertension, diabetes, smoking, BMI, total cholesterol, estimated GFR, prior MI, prior coronary revascularization, beta-blocker use, lipid-lowering therapy, and randomized treatment arm |
| PROSPER 2010 | 1.age, sex, smoking status, assay conditions and country，study treatment (placebo or pravastatin), systolic blood pressure, history of diabetes, MI, history of hypertension, LDL cholesterol, HDL cholesterol, triglycerides, log CRP and WCC，history of vascular disease at baseline（adjust for HR for quartiles）.  2.Age ,Sex, Smoker ,Systolic BP ,Diabetes ,BMI ,Hypertension , LDLc ,HDLc ,Triglyceride ,Log CRP ,WCC ，Pravastatin treatment（adjust for HR for per 1SD）. |
| PROVE IT-TIMI22 2006 | age, index diagnosis, prior MI, prior renal impairment, diabetes mellitus, treatment arm, baseline LDL, and baseline CRP. |
| Rotterdam 2005 | age, sex, body mass index, systolic blood pressure, non-HDL cholesterol level, HDL cholesterol level, diabetes, smoking, cholesterol-lowering  medication, CRP, white blood cell count, and alcohol consumption |
| SPARCL trial 2017 | age, sex, race[white/nonwhite], treatment group, smoking status, diabetes mellitus, systolic blood pressure, hypertension treatment, high-density lipoprotein cholesterol, apolipoprotein A1, entry event [stroke or TIA], time since entry event, and geographic region |
| STABILITY trial 2016 | randomized treatment, geographic region, age, sex, body mass index, current smoking, hypertension, diabetes mellitus, prior MI, prior coronary revascularization, multivessel coronary heart disease, polyvascular disease, significant renal dysfunction, routine biochemical variables (hemoglobin, white blood cell count, estimated glomerular filtration rate [Chronic Kidney Disease Epidemiology Collaboration], low-density lipoprotein cholesterol, high-density lipoprotein cholesterol, and triglycerides), and prognostic biomarkers (N-terminal proB-type natriuretic peptide, high-sensitivity cardiac troponin T, cystatin C, high-sensitivity C-reactive protein, and interleukin 6. |
| WHI-OS 2012 | age, race/ethnicity, prior diabetes, angina, statin use, current smoking, the natural logs of systolic blood pressure, total and high-density lipoprotein cholesterol and C-reactive protein, family history of premature myocardial infarction, and hemoglobin A1c among diabetics, current and past use of hormone therapy as appropriate. |
| VA-HIT 2008 | age, hypertension, BMI, diabetes, active smoking, baseline values of LDL-C, HDL-C, triglycerides, and CRP. |

ARIC: Atherosclerosis Risk in Communities study; Bruneck: The Bruneck study; BLSA: Beijing Longitudinal Study of Aging; CHANCE : Clopidogrel in High-Risk Patients with Acute Non-disabling Cerebrovascular Events) trial;CHS: Cardiovascular Health Study; FOS: Framingham Offspring Study; HPS:Heart Protection Study; MDCS: Malmö Diet and Cancer Study;NOMAS: Northern Manhattan Study; NPHS-II: Northwick Park Heart Study II;PEACE: Prevention of Events with Angiotensin Converting Enzyme Inhibition; PROSPER: Prospective Study of Pravastatin in the Elderly at Risk; PROVEIT-TIMI 22: PRavastatin Or ator Vastatin Evaluation and Infection Therapy – Thrombolysis In Myocardial Infarction 22; Rotterdam: The Rotterdam study;WHI-HaBPS: Women’s Health Initiative - Hormones and Biomarkers Predicting Stroke in Women; WHI-OS: Women's Health Initiative Observational Study

**Appendix Supplement 5.** Assessment of publish bias of included studies by funnel plot and Egger’s test in studies

**Figure S-1.**Begg's rank correlation test (p= 0.902) and Egger's linear regression test (p= 0.845) for total stroke with 1 SD higher Lp-PLA2 activity.

**Figure S-2.** Begg's rank correlation test (p= 0.024) and Egger's linear regression test (p= 0.331) for total strokes with 1 SD higher Lp-PLA2 mass.

**Figure S-3.** Begg’s test (*P* = 0.276) and Egger’s test (*P* = 0.080) for total strokes with highest vs lowest Lp-PLA2 activity.

**Figure S-4.** Begg's rank correlation test (p = 1.000) and Egger's linear regression test (p= 0.970) for ischemic stroke with 1 SD higher Lp-PLA2 activity.

**Figure S-5.**  Begg's rank correlation test (p = 0.133) and Egger's linear regression test (p= 0.171) for ischemic stroke with 1 SD higher Lp-PLA2 mass

.

| **Appendix Supplement 6.** Table S-1. Subgroup Analyses of the Association between Lp-PLA2 and Risk of Stroke | | | | | | | | |  |
| --- | --- | --- | --- | --- | --- | --- | --- | --- | --- |
| Subgroup | Per SD change of Lp-PLA2 activity | | | | | | |  | |
|  | Number of studies | RR (95% CI) | | | | | P/I^2^ value* |  | |
| Gender(all stroke) |  |  | | |  | | 0.29/18.7% |  | |
| Both gender | 13 | 1.08 | | | (1.03, 1.15) | |  |  | |
| Men | 3 | 1.04 | | | (0.71, 1.51) | |  |  | |
| Momen | 2 | 0.98 | | | (0.88, 1.10) | |  |  | |
|  |  |  | | |  | |  |  | |
| Mean age(years) |  |  | | |  | |  |  | |
| All stroke |  |  | | |  | | 0.91/0% |  | |
| ≥65 | 6 | 1.08 | | | (0.97, 1.20) | |  |  | |
| <65 | 11 | 1.07 | | | (1.01, 1.13) | |  |  | |
| Ischemic stroke |  |  | | |  | | 0.96/0% |  | |
| ≥65 | 4 | 1.09 | | | (0.93, 1.27) | |  |  | |
| <65 | 6 | 1.08 | | | (1.01, 1.16) | |  |  | |
|  |  |  | | |  | |  |  | |
| Study design (all stroke) | | |  |  | | 0.93/0% | | |  |
| Prospective cohort study | 10 | 1.08 | | | (0.99, 1.17) | |  |  | |
| RCT | 7 | 1.07 | | | (1.02, 1.13) | |  |  | |
|  |  |  | | |  | |  |  | |
| follow-up period(years)(all stroke) | | | |  | | 0.60/0% | | |  |
| ≥10 | 7 | 1.10 | | | (0.98, 1.22) | |  |  | |
| <10 | 10 | 1.06 | | | (1.01, 1.12) | |  |  | |
|  |  |  | | |  | |  |  | |
| Inclusion of participants with baseline CVD (yes vs. no) (all stroke) | | | | | | 0.86/0% | | |  |
| Yes | 5 | 1.08 | | | (0.99, 1.18) | |  |  | |
| No | 12 | 1.07 | | | (1.01, 1.14) | |  |  | |
|  |  |  | | |  | |  |  | |
| With CVD type in baseline (all strokes) | | | |  | | 0.15/52.1% | | |  |
| Cerebral vascular disease | 2 | 1.16 | | | (1.05, 1.29) | |  |  | |
| CHD | 4 | 1.04 | | | (0.94, 1.15) | |  |  | |
|  | | | | | | | | | |
| NOS quality scores (all stroke) | | |  |  | | 0.94/0% | | |  |
| ≥8 stars | 13 | 1.08 | | | (1.02, 1.14) | |  |  | |
| <8 stars | 4 | 1.04 | | | (0.93, 1.17) | |  |  | |
|  |  |  | | |  | |  |  | |
| Ischemic stroke events sizes ( ischemic stroke) | | | |  | | 0.74/0% | | |  |
| n≥200 | 5 | 1.06 | | | (1.01, 1.12) | |  |  | |
| n<200 | 5 | 1.10 | | | (0.92, 1.33) | |  |  | |

Note . * P/I^2^ value of test for subgroup differences.

**Appendix Supplement 7** Subgroup analyses based on per SD change of Lp-PLA2 activity


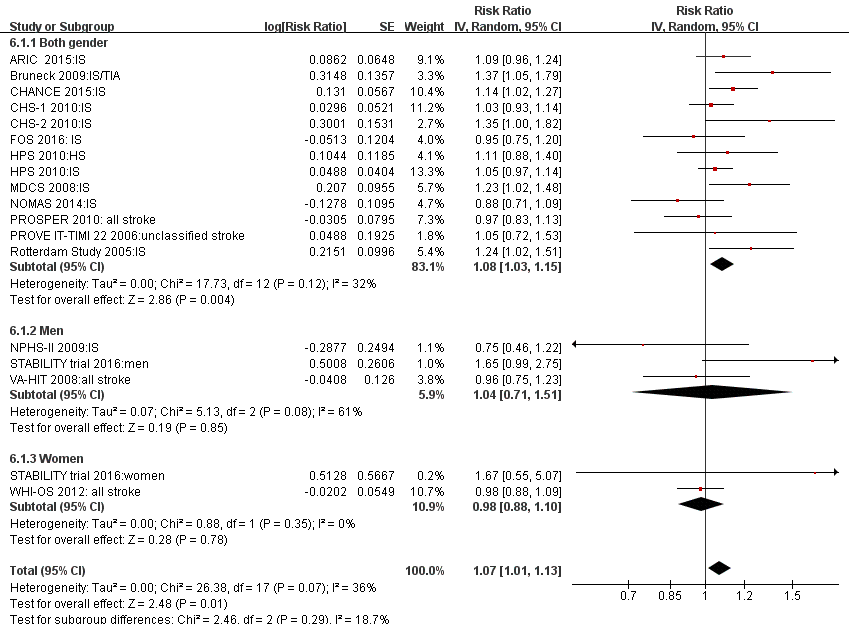


**Figrure S-6.** Subgroup analysis of RR and 95% CI of all stroke and lp-pla2 activity 1 SD change by gender.

**
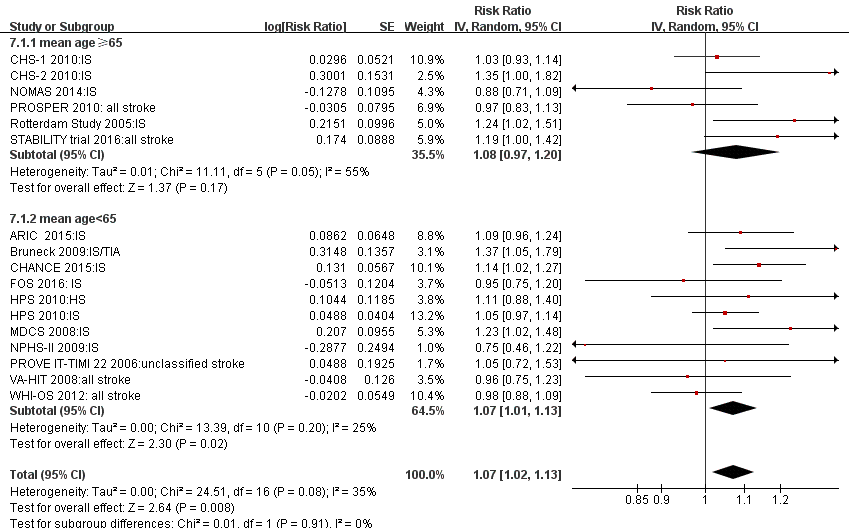
**

**Figrure S-7.** Subgroup analysis of RR and 95% CI of overall stroke and lp-pla2 activity 1 SD change by mean age(≥65 vs <65 years)

**
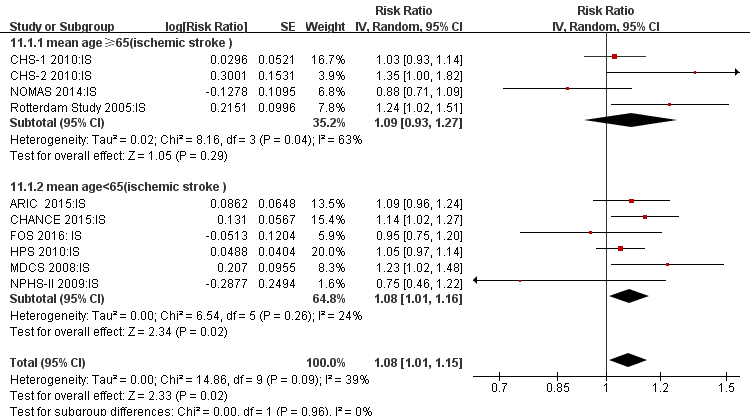
**

**Figrure S-8.** Subgroup analysis of RR and 95% CI of ischemic stroke and lp-pla2 activity 1 SD change by mean age(≥65 vs <65 years)

**
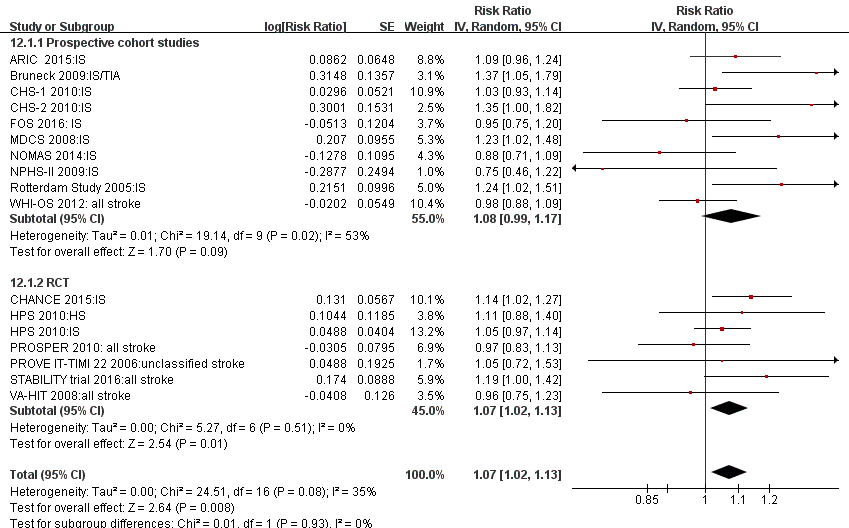
**

**Figrure S-9.** Subgroup analysis of RR and 95% CI of all strokes and lp-pla2 activity 1 SD change by study design (prospective cohort studies vs RCT )

**
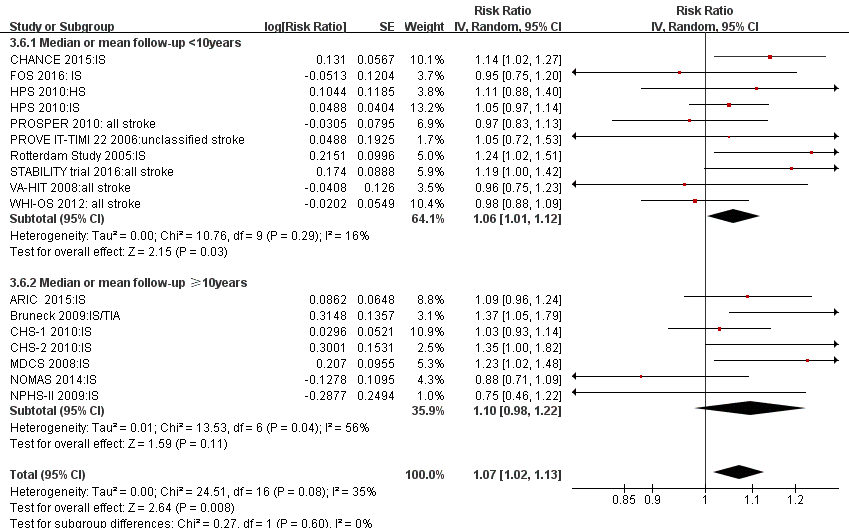
**

**Figrure S-10.** Subgroup analysis of RR and 95% CI of all strokes and lp-pla2 activity 1 SD change by median or mean follow-up (median or mean follow-up ≥10years vs median or mean follow-up <10years )

**
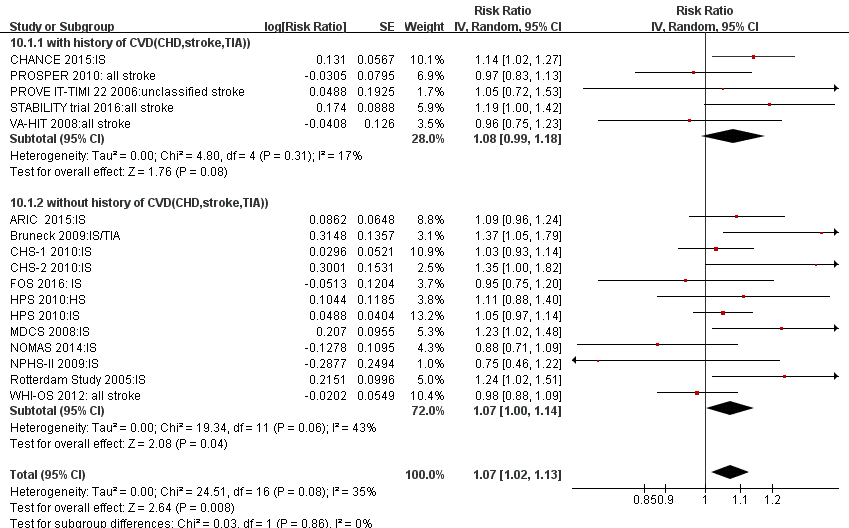
**

**Figrure S-11.** Subgroup analysis of RR and 95% CI of all strokes and lp-pla2 activity 1 SD change by inclusion of participants with baseline CVD (yes *vs.* no)


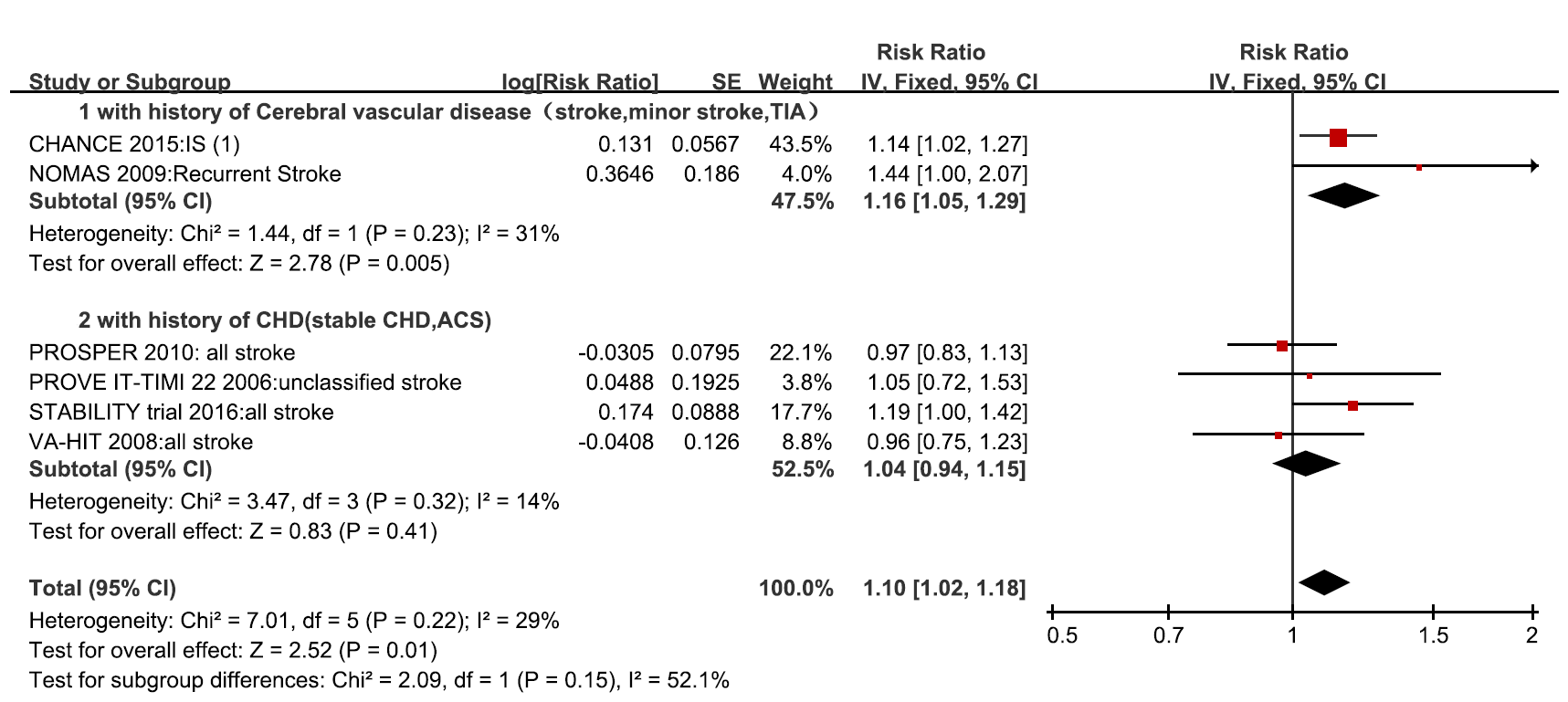


**Figrure S-12.** Subgroup analysis of RR and 95% CI of all strokes and lp-pla2 activity 1 SD change by CVD type (Cerebral vascular disease *vs.* CHD)

**
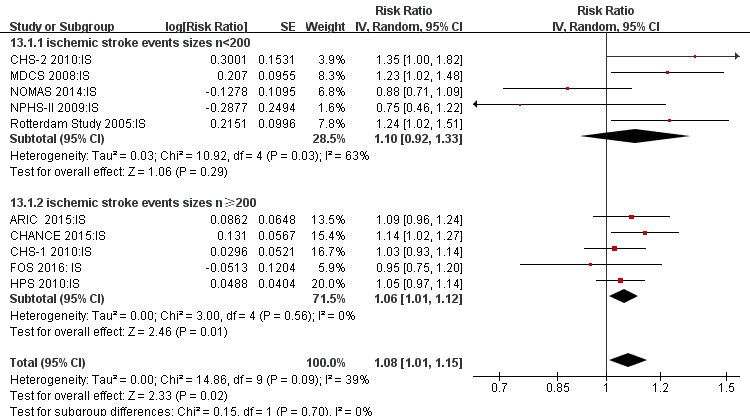
**

**Figrure S-13.** Subgroup analysis of RR and 95% CI of ischemic stroke and lp-pla2 activity 1 SD change by ischemic stroke events sizes (n≥200 *vs.* n<200)

**
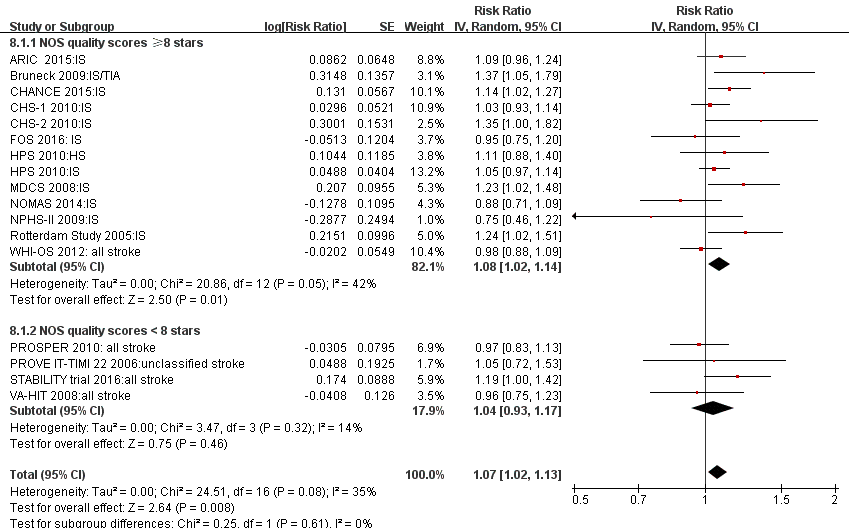
**

**Figrure S-14.**  Subgroup analysis of RR and 95% CI of all strokes and lp-pla2 activity 1 SD change by NOS quality scores (≥8 stars *vs.* <8 stars)

**Appendix Supplement 8.** Forest plot of Pooled RR and 95%CI for CVSD with 1 SD higher Lp-PLA2 activity


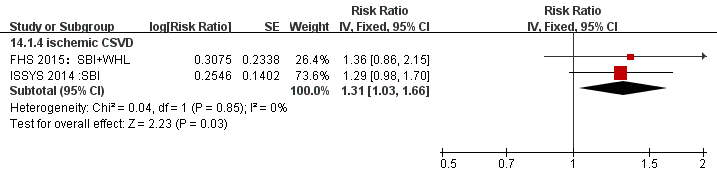


**Figrure S-15**. Pooled RR and 95%CI for CVSD with 1 SD higher Lp-PLA2 activity
